# Interactions between genes involved in the antioxidant defence system and breast cancer risk

**DOI:** 10.1038/sj.bjc.6603272

**Published:** 2006-07-25

**Authors:** M Z Oestergaard, J Tyrer, A Cebrian, M Shah, A M Dunning, B A J Ponder, D F Easton, P D P Pharoah

**Affiliations:** 1 Department of Public Health and Primary Care, Strangeways Research Laboratories, Cambridge CB1 8RN, UK; 2 Cancer Research UK Department of Oncology, Strangeways Research Laboratories, Cambridge CB1 8RN, UK; 3 Cancer Research UK Genetic Epidemiology Group, Strangeways Research Laboratories, Cambridge CB1 8RN, UK

**Keywords:** epistasis, gene–gene interactions, breast cancer, antioxidant defence

## Abstract

The aim of the study is to examine the association between multilocus genotypes across 10 genes encoding proteins in the antioxidant defence system and breast cancer. The 10 genes are *SOD1*, *SOD2*, *GPX1*, *GPX4*, *GSR*, *CAT*, *TXN*, *TXN2*, *TXNRD1* and *TXNRD2*. In all, 2271 cases and 2280 controls were used to examine gene–gene interactions between 52 single nucleotide polymorphisms (SNPs) that are hypothesised to tag all common variants in the 10 genes. The statistical analysis is based on three methods: unconditional logistic regression, multifactor dimensionality reduction and hierarchical cluster analysis. We examined all two- and three-way combinations with unconditional logistic regression and multifactor dimensionality reduction, and used a global approach with all SNPs in the hierarchical cluster analysis. Single-locus studies of an association of genetic variants in the antioxidant defence genes and breast cancer have been contradictory and inconclusive. It is the first time, to our knowledge, the association between multilocus genotypes across genes coding for antioxidant defence enzymes and breast cancer is investigated. We found no evidence of an association with breast cancer with our multilocus approach. The search for two-way interactions gave experiment-wise significance levels of *P*=0.24 (*TXN* [t2715c] and *TXNRD2* [g23524a]) and *P*=0.58 (*GSR* [c39396t] and *TXNRD2* [a442g]), for the unconditional logistic regression and multifactor dimensionality reduction, respectively. The experiment-wise significance levels for the three-way interactions were *P*=0.94 (*GPX4* [t2572c], *TXN* [t2715c] and *TXNRD2* [g23524a]) and *P*=0.29 (*GSR* [c39396t], *TXN* [t2715c] and *TXNRD2* [a442g]) for the unconditional logistic regression and multifactor dimensionality reduction, respectively. In the hierarchical cluster analysis neither the average across four rounds with replacement of missing values at random (*P*=0.12) nor a fifth round with more balanced proportion of missing values between cases and controls (*P*=0.17) was significant.

Breast cancer is the most common malignancy affecting women worldwide. It is estimated to constitute 18% of all female cancers with more than one million incident cases worldwide every year (McPherson *et al*, 2000). Several epidemiological studies have shown that the risk of breast cancer is increased for women with a family history of the disease (Pharoah *et al*, 1997; Collaborative Group on Hormonal Factors in Breast Cancer, 2001; Dite *et al*, 2003), and twin studies have suggested that most of the excess familial risk of breast cancer is due to inherited genetic factors (Lichtenstein *et al*, 2000). However, the known breast cancer susceptibility genes can account for only 20–25% of the excess familial risk (Easton, 1999). Low-penetrance alleles are hypothesised to be fairly common and expected to contribute substantially to breast cancer incidence (Pharoah *et al*, 2004).

The molecular mechanisms underlying the aetiology of breast cancer are not fully understood. It is, however, generally thought that the initiation of breast cancer occurs after an accumulation of genetic alterations that result in either activation of oncogenes and/or inactivation of tumour suppressor genes. These lead to either cellular proliferation and/or abnormal programmed cell death. Reactive oxygen species (ROS) can damage DNA in the form of mutations, deletions, gene amplification and rearrangements. These changes may cause initiation of programmed cell death, or activation of several proto-oncogenes and/or inactivation of some tumour suppressor genes. Reactive oxygen species can also cause lipid peroxidation, protein alterations and/or damage to the mitochondria (Hayes and McLellan 1999; Mates and Sanchez-Jimenez 2000; Ray and Husain 2002). Reactive oxygen species include the superoxide anion (O_2_−), hydrogen peroxide (H_2_O_2_) and the hydroxyl radical (^*^OH). These are constantly generated in the cell as a result of aerobic metabolism and can also be generated as a result of inflammation, cellular stress and from the metabolism of exogenous compounds. They serve important cellular functions at normal concentrations. However, high and/or sustained levels of ROS, referred to as oxidative stress, are suggested to be associated with several diseases, including cancer (Mates and Sanchez-Jimenez, 2000).

A defence system exists to combat ROS and to secure a redox-balance where oxidants are kept at nontoxic levels. This defence system is composed of both nonenzymatic and enzymatic compounds. The nonenzymatic compounds include flavonoids, gluthathione and antioxidant vitamins such as vitamin A, C and E (Mates and Sanchez-Jimenez, 2000). The enzymatic defence system of ROS is called the antioxidant defence system (ADS). The major factors in ADS are superoxide dismutases (SOD), catalase (CAT) and gluthathione peroxidase (GPX) (Hayes and McLellan, 1999; Mates and Sanchez-Jimenez, 2000; Ray and Husain, 2002; Townsend *et al*, 2003) Antioxidant enzymes can inhibit the initiation of carcinogenesis, affect tumour progression, and their expression is reduced in many types of cancerous cells (Oberley and Oberley, 1997; Li *et al*, 2000; Mates and Sanchez-Jimenez, 2000).

The thioredoxin proteins (TRX1 and TRX2) also serve important redox-catalytic functions in the cell. TRX1 is the most studied and the function of TRX2 remains unknown. TRX1 supplies reducing equivalents to thioredoxin peroxidases and ribonucleotide reductase, regulates transcription factors and enzyme activity, stimulates cell growth and is an inhibitor of apoptosis. There is an increased level of TRX1 in many cancerous cells and the protein is associated with aggressive tumour growth (Powis *et al*, 2000; Powis and Montfort, 2001). Thioredoxin reductase is the only known class of enzymes that can reduce TRX1 and TRX2. Only two confirmed forms of thioredoxin reductaser exist, TRXR1 and TRXR2. TRXR1 and TRXR2 catalyse several reductions of both exogenous and endogenous compounds and they may play a role in the protection against cell growth and cell transformation (Mustacich and Powis, 2000).

Most candidate-gene association studies investigating the association between genetic variants in the genes coding enzymes involved in antioxidant defence and breast cancer have only studied a single SNP in one of the two genes, *SOD2* and *GPX1*. The results have been inconsistent and inconclusive. We have recently investigated the single-locus effects of common variants in 10 genes (*SOD1*, *SOD2*, *GPX1*, *GPX4*, *GSR*, *CAT*, *TXN*, *TXN2*, *TXNRD1* and *TXNRD2*) in a large breast cancer case–control study. We used 52 SNPs to tag common variants in the genes and found marginal evidence for association with polymorphisms in *CAT (*g27168a) (*P*=0.039), *TXN* (t2715c) (*P*=0.007) and *TXNRD2* (g23524a) (*P*=0.040), results that were no longer significant after correcting for multiple testing (Cebrian *et al*, 2006). However, the absence of significant single-locus main effects does not exclude the possibility of important interactions between the loci. Studies of genetic epistasis in model organisms suggest that epistasis makes a large contribution to the genetic regulation of complex traits in various organisms, and in animal models there are several examples of two-locus epistatic interactions that are not associated with detectable single-locus effects (Carlborg and Haley, 2004). Furthermore, in the context of human genetic association studies it has been shown that it is possible to detect epistatic interactions in the absence of single-locus main effects under several plausible genetic models (Ritchie *et al*, 2001; Marchini *et al*, 2005). The purpose of this study was to search for evidence for interactions between common variants in genes involved in the antioxidant defence system using three complementary approaches.

## MATERIALS AND METHODS

### Cases and controls

The selection and characteristics of cases and controls are described in detail in Cebrian *et al*, 2006. In brief, we used genotype data for 52 SNPs chosen to tag the known common variation in 10 genes of interest (*SOD1*, *SOD2*, *GPX1*, *GPX4*, *GSR*, *CAT*, *TXN*, *TXN2*, *TXNRD1* and *TXNRD2*) from a case-control study of 2271 cases and 2280 controls (all subjects are women). We used data from the International HapMap Project and resequencing data from the Environmental Genome Project to identify tagging SNPs. We aimed to tag all variants with minor allele frequency (MAF) >0.05 with a correlation of 0.8 or greater (see Cebrian *et al* for details). A list of the SNPs assayed is given in Table 1. All cases had been diagnosed with invasive breast cancer and were ascertained through the East Anglian Cancer Registry as part of an ongoing population-based study called SEARCH (breast). Controls were randomly selected from the Norfolk arm of the European Prospective Investigation of Cancer. The ethnic background of both cases and controls is similar with >98% white. Table 2 summarises characteristics of the cases. Cases are younger than controls. The median ages were 51 and 65, and the interquartile ranges were (45–55) and (59–71), for cases and controls, respectively. Among cases, 69% were incident cases. The morphology and histopathological grade or clinical stage were similar for incident and prevalent cases. The study is approved by the Eastern Region Multicentre Research Ethics Committee, and all patients gave written informed consent.

All samples were genotyped for selected tagSNPs using the ABI PRISM 7900 sequence detection system or ‘Taqman’ (Applied Biosystems). Cases and controls were arrayed together in 12 384-well plates and a 13th plate contained eight duplicate samples from each of the 12 plates to ensure a good quality of genotyping. The concordance was >99% for all SNPs. Failed genotypes were not repeated. The rate for failed genotype did not exceed 8.3% for any of the SNPs.

### Statistical methods

We used three different approaches to look for epistasis, all of which have been described previously: one parametric method–unconditional logistic regression (Marchini *et al*, 2005); and two non-parametric methods–multifactor dimensionality reduction (Ritchie *et al*, 2001) and hierarchical cluster analysis (Hastie *et al*, 2001; Levenstein *et al*, 2003). Unconditional logistic regression and multifactor dimensionality reduction were programmed in C++. Stata version 8.2 was used to program the hierarchical cluster analysis.

All two- and three-way SNP combinations were examined with unconditional logistic regression and multifactor dimensionality reduction, while a global approach with all SNPs was used in the hierarchical cluster analysis. The number of possible *d*-way combinations out of *n* tagSNPs is given by: *n*!/(*d*!^*^(*n−d*)!). Hence, the number of possible two-way interactions (*n*=52, *d*=2) is 1326 and the number of three-way interactions (*n*=52, *d*=3) is 22 100.

For all three analyses methods the problems of multiple testing and calculation of an experiment-wise significance are addressed by permutation testing. The most significant test statistic derived from the original data set is compared to an empirical null distribution of the test statistic, which is created by permuting or shuffling the labels of cases and controls. The assumption is that the shuffling of the case–control label will break any possible association between genotype and the disease while maintaining the correlation structure of the genetic markers. The proportion of permutation samples in which the test statistic is at least as significant as the test statistic in the original data set is the significance level. The number of permutations was chosen according to the required accuracy of the evidence for association with breast cancer (minimum of 100) and computational intensity.

The logistic regression method (ULR) aims to find combinations of SNPs that increase the risk of developing breast cancer compared to no effect of the SNPs. For each combination of SNPs a likelihood ratio test is conducted where a saturated logistic model (with all main and interaction effects) is compared to the null model (with no main or interaction effects). The null model has one degree of freedom and a saturated model for n number of loci has 3^*n*^ degrees of freedom. The algorithm used reduces the number of degrees of freedom by *m* (compared to the theoretical maximum) in a contingency table when *m* cells are empty in both cases and controls. The likelihood ratio test is calculated as: 2(log *Lik*_full_−log *Lik*_null_)∼χ_df_^2^ and the test statistics are assumed to follow a *χ*^2^ distribution with 8 degrees of freedom (df) for the two-locus interaction and 26 df for the three-locus interactions. The different combinations of SNPs are indirectly compared through their likelihood ratio test with the null model. The most significant *P*-value from the likelihood ratio tests is used in the permutation test with 1000 permutations for both the two-way and three-way models.

The multifactor dimensionality reduction (MDR) method was introduced by Ritchie *et al*, in 2001. The aim of MDR is to reduce the number of dimensions in the analysis to one by using a model for cases and controls that classifies multilocus genotypes into either a high- or low-risk group. This grouping depends on the ratio of cases to controls for each genotype (the version of the MDR method used in the analysis assumes an equal number of cases and controls, which is not required in later versions of the method). If the ratio is ⩾1, the group is a high-risk group and low-risk group if the ratio is <1. For model validation both split-sample and cross-validation is used. The data is divided into 10 equal-sized data sets. In all, 9/10 of the data, called the training set, is used to develop a model and the remaining 1/10 of the data, called the test set, is considered an independent data set and used to test the internal validity of the model by predicting the grouping of genotypes into high- and low-risk groups. Cross-validation is applied to protect against chance division of the data set from the split-sample and is used to calculate the average internal prediction error. Hypothesis testing was based on the average prediction error, which is used in the permutation testing with 100 permutations.

We used agglomerative or bottom-up hierarchical cluster analysis (HCA). The purpose of HCA is to categorise individuals into groups (clusters) according to the similarity of their genotype over all 52 loci independent of case–control status. Within a cluster individuals are more similar to each other than to an individual outside the cluster. The algorithm builds up a hierarchical structure of clusters with one individual in each cluster at the lowest level and one cluster containing all individuals at the highest level. The similarity-measure applied was based on the number of SNPs for which individuals have the same genotype. Complete linkage agglomerative clustering was used to build up groups, where the intergroup dissimilarity is defined as the dissimilarity between the most dissimilar pair of individuals with one person from each cluster. The dissimilarity within a cluster is monotonically increasing as more and more clusters are merged. We then used several levels of the hierarchy to generate between 2 and 10 clusters. Association of group membership with case–control status was assessed using standard *χ*^2^ tests. Each test statistic is calculated from a 2 × *k* contingency table, with cases and controls as rows and *k* columns, which represent the number of clusters at each step. *k* is thus between 2 and 10. The smallest of these *P*-values is chosen to calculate the experiment-wise significance by permutation testing with 10 000 permutations.

The proportion of missing values was 6.2% for cases and 1.8 % for controls. It is unlikely that missing values are related to genotype, but the problem of missing data was handled differently for each of the analytic methods in order to minimise the chance of bias. For the logistic regression, samples with missing genotypes were excluded. This will not result in bias assuming missing values are not related to genotype. However, the multifactor dimensionality reduction will tend to give more significant results if the distribution of the missing values is differential between cases and controls, as it assumes one case for every control. Therefore, to balance the proportion of missing values between cases and controls for this method, any subject was excluded if they had more than 17 missing genotypes. This resulted in the exclusion of 316 subjects from the data (26 controls and 290 cases) and left 0.91% missing values for cases and 1.4% for controls. The threshold of missing values was chosen to minimise the number of excluded subjects and to find a balanced division of missing values between cases and controls. As all genotypes were used for the hierarchical cluster analysis all subjects were included, but the missing values were replaced at random based on the distribution of genotypes in the nonmissing data.

## RESULTS

Tables 3 and 4 outline the 10 SNP combinations with smallest *P*-values for the two- and three-way interactions identified by logistic regression. The most significant combinations of loci were, for the two-way combination, TRX1-14 and TRXR2-04 (naïve *P*=0.00026) and, for the three-way combination, GPX4-02, TRX1-14 and TRXR2-04 (naïve *P*=0.00021). Neither of these was significant in the permutation analysis (*P*=0.24 and *P*=0.94, respectively). Table 5 outlines the multilocus genotype frequencies and effect sizes for the most significant SNP combination identified with logistic regression (TRX1-14 and TRXR2-04). The SNPs TRX1-14 and TRXR2-04 are both intronic and unlikely to have a direct functional effect. GPX4-02 is located in the 3′ untranslated region and is a potential regulatory variant that affects gene expression.

Tables 6 and 7 illustrate the 10 combinations with lowest classification error for the whole data set for the two- and three-way interactions, respectively, for the multifactor dimensionality reduction. The combinations with lowest average prediction error were for the two-way interaction GSR-16 and TRXR2-05; and the three-way interaction GSR-16, TRX1-14 and TRXR2-05. The experiment-wise significance estimated by permutation testing showed that the average prediction errors were not better than expected by chance for the best of either the two-way (*P*=0.58) or the three-way combinations (*P*=0.29). Table 8 outlines the multilocus genotype frequencies and effect sizes for the most significant SNP combination identified with multifactor dimensionality reduction (GSR-16 and TRXR2-05). The SNPs GSR-16, TRXR2-05 and TRX1-14 are all intronic.

The method of hierarchical cluster analysis was very unstable as results varied a lot with different random number seeds used to replace the missing values at random. Thus, four different random number seeds were used and the average significance level was *P*=0.12. In addition, a fifth round of the hierarchical cluster analysis was conducted where subjects were ignored if they had more than 17 missing values. This way the proportion of missing values was more balanced between cases and controls. The result from the fifth round gave a *P*-value of 0.17.

## DISCUSSION

We have found no evidence that interactions between common variants in 10 genes involved in the antioxidant defence system are associated with breast cancer risk, based on data from over 2000 cases and 2000 controls. Despite the comparatively large sample size, statistical power of this study may be limited. For specific two- and three-way combinations scarcity of data in individual cells in the contingency table may render risk estimates unreliable. This problem is compounded by the issue of multiple testing as there are 1326 possible two-way and 22 100 possible three-way combinations, and so only very highly significant interactions would remain significant after allowing for multiple testing by permutation analysis. Indeed, the naïve *P*-value for most significant two-way interaction in the logistic regression analysis of 0.00026 became nonsignificant (*P*=0.24) after permutation testing. We estimated that a naïve *P*-value of 0.000044 would be needed for significance at the 5% level for the two-way logistic regression. It would be difficult to determine the exact power of the methods used because this would depend on the underlying genetic model, which is unknown. However, to give some feel for the sort of effects that would be detectable Table 9 illustrates the multilocus genotype frequencies and effect sizes for a permuted data set that was significant at the 5% threshold for the two-way logistic regression. Furthermore, Marchini *et al* (2005) simulated data for 300 000 loci in a similar sized case–control study to ours (2000/2000) under plausible scenarios for epistatic interaction and showed that the logistic regression method has reasonable power to detect gene–gene interactions even in the absence of main effects and with conservative correction for multiple testing.

Possible problems of confounding and bias must also be considered. However, most plausible biases would be expected to be away from the null and result in false positive associations. For example, stratification due to hidden population substructure is often cited as a potential problem in genetic association studies, but is unlikely to have been important in this analysis–we have previously looked for association between unlinked markers in the controls and found no evidence for population stratification (Goode *et al*, 2005). Similarly, differential measurement of genotype between cases and controls is unlikely to be important as, if present, this would be expected to result in a false positive result. On the other hand, nondifferential genotyping errors would have diluted a possible association. Given that genotyping assays are only accepted if there is 100% concordance between duplicate samples, the number of genotyping errors is expected to be trivial.

Missing data is more likely to have introduced bias as there were more missing values in cases than in controls. However, unless the missing genotypes were not random with respect to genotype category and related to case–control status, missing data will not bias the ULR. The loss of data will result in a small loss of statistical power. Missing data are more likely to be important for MDR, which relies on a balanced case–control design, but as the result was negative, it seems unlikely that the adjustment used for missing values was inadequate. For the HCA the distribution of values in the nonmissing data was used to replace missing values at random, but the analysis was very sensitive to the different random number seeds. The estimate in the fifth round of HCA with balanced proportion of missing values between cases and controls might therefore be more appropriate.

The lack of interaction between the SNPs analysed in this study does not exclude the presence of other important interacting variants in the 10 genes. A comprehensive SNP tagging approach was used in an attempt to capture all the known common variation in the genes under study, but this process is imperfect. Common variants in the genes *SOD2*, *CAT*, *GPX1*, *GPX4*, *GSR* and *TXN* were identified by resequencing in a modest sample of individuals (90 in total and 62 non-African Americans). Consequently, some common SNPs in these genes will have been missed by chance. Nevertheless, the high SNP density in the genes with 0.21–0.73 kbp per SNP is very likely to be sufficient to tag any SNP missed by resequencing. Resequencing data were not available for the genes *SOD1*, *TXN*2*, TXNRD1* and *TXNRD2* and data from the HapMap project were used to select tagSNPs. As coverage of the HapMap data for these genes was at a SNP density of 2–4 kbp per SNP it is anticipated that any further SNPs will be well tagged. It is also possible that the populations used for selecting tagSNPs–mixed American ethnicities for resequencing data after exclusion of African Americans and CEPH trios for HapMap–do not adequately represent the population from which our study has been drawn. However, the haplotype frequencies estimated in our controls are similar to those estimated using both EGP and HapMap data. Finally, the tagging approach used is unlikely to adequately capture rare variants (minor allele frequency <0.05), and so the possibility that there are important rare susceptibility variants acting together cannot be excluded.

It is also possible that common variants in these genes interact with variants in genes in other relevant biological pathways. For example, studies of modular epistasis in yeast metabolism suggest that epistasis extend beyond functional modules of genes and frequently involves interactions between, rather than within, functional modules (Segre *et al*, 2005).

We have shown that it is straightforward to implement several methods to search for gene–gene interactions in a moderately large data set, but the question of which method is superior can only be answered when real gene–gene interactions are identified in human disease. Our analysis was restricted to the study of variation within a single cellular pathway, but analysis of common variants across the whole genome may prove to be more fruitful. It is likely that data from several genome-wide association studies will be available for breast cancer, as well as other phenotypes, and the use of multiple analytic methods will be appropriate for comparative purposes. Advances in the field of systems biology may help to reduce the genomic search space in both candidate gene and genome-wide association studies (Irizarry *et al*, 2005).

## Figures and Tables

**Table 1:** Details of SNPs used in the analyses

**Gene**	**Label**	**db SNP^a^**	**MAF^b^**	**Location**	**Codon number**	**Amino acid^c^**
CAT	CAT-01	rs1001179	0.22	Regulatory	0	
CAT	CAT-03	rs769217	0.22	Silent	389	D>D
CAT	CAT-04	rs511895	0.42	Intronic	0	
CAT	CAT-05	rs7104301	0.26	Regulatory	0	
CAT	CAT-08	rs1049982	0.32	5′UTR^d^	0	
GPX1	GPx1-01	rs3448	0.25	Intergenic		
GPX4	GPx4-02	rs713041	0.46	3′UTR	0	
GPX4	GPx4-04	rs4807542	0.16	Silent	12	P>P
GPX4	GPX4-06	rs757229	0.46	Intergenic	0	
GSR	GSR-01	rs1002149	0.16	Regulatory	0	
GSR	GSR-07	rs4628224	0.19	Intronic	0	
GSR	GSR-12	rs8190924	0.05	Intronic	0	
GSR	GSR-14	rs2978663	0.37	Intronic	0	
GSR	GSR-16	rs8191009	0.20	Intronic	0	
GSR	GSR-18	rs3926402	0.39	Intronic	0	
GSR	GSR-19	rs2911678	0.22	Intronic	0	
SOD1	SOD1-01	rs4998557	0.12	Intronic	0	
SOD1	SOD1-02	rs202445	0.17	Regulatory	0	
SOD1	SOD1-03	rs2070424	0.07	Intronic		
SOD2	SOD2-02	rs1799725	0.49	Missense	16	V>A
SOD2	SOD2-05	rs2842958	0.21	Intronic	0	
TXN	TRX1-02	rs1410051	0.24	Intronic	0	
TXN	TRX1-03	rs4135168	0.26	Intronic	0	
TXN	TRX1-05	rs4135192	0.33	Intronic	0	
TXN	TRX1-06	rs2418076	0.27	Intronic	0	
TXN	TRX1-07	rs4135208	0.33	Intronic	0	
TXN	TRX1-08	rs4135211	0.07	Intronic	0	
TXN	TRX1-09	rs4135225	0.33	Intronic	0	
TXN	TRX1-10	rs2776	0.50	3′UTR	0	
TXN	TRX1-11	rs2301241	0.41	5′UTR	0	
TXN	TRX1-12	rs4135165	0.09	Intronic	0	
TXN	TRX1-13	rs4135172	0.08	Intronic	0	
TXN	TRX1-14	rs4135179	0.23	Intronic	0	
TXN	TRX1-15	rs4135215	0.16	Intronic	0	
TXN	TRX1-16	rs4135221	0.13	Intronic	0	
TXN2	TRX2-01	rs2281082	0.19	Intronic	0	
TXN2	TRX2-03	rs8139906	0.18	Intronic	0	
TXN2	TRX2-04	rs8140110	0.09	Intronic	0	
TXNRD1	TRXR1-01	rs4964778	0.18	Intronic	0	
TXNRD1	TRXR1-02	rs4964779	0.12	Intronic	0	
TXNRD1	TRXR1-03	rs4564401	0.07	Intronic	0	
TXNRD1	TRXR1-04	rs10861201	0.23	Intronic	0	
TXNRD2	TRXR2-01	rs2073752	0.28	Missense	370	T>I
TXNRD2	TRXR2-03	rs5748469	0.33	Missense	66	S>A
TXNRD2	TRXR2-04	rs756661	0.47	Intronic	0	
TXNRD2	TRXR2-05	rs2020917	0.28	Intronic	0	
TXNRD2	TRXR2-07	rs740603	0.46	Intergenic	0	
TXNRD2	TRXR2-08	rs4485648	0.20	Intronic	0	
TXNRD2	TRXR2-09	rs732262	0.09	Intronic	0	
TXNRD2	TRXR2-10	rs1548357	0.27	Intronic	0	
TXNRD2	TRXR2-11	rs2073750	0.21	Intronic	0	
TXNRD2	TRXR2-12	rs3788306	0.30	Intronic	0	

a db: rs number for each SNP which uniquely identifies it.

b MAF: minor allele frequency.

c Labels for amino acids: aspartic acid (D); proline (P); valine (V); alanine (A); threonine (T); isoleucine (I); serine (S).

d UTR: untranslated region.

**Table 2:** Characteristics of cases

		**Morphology**			**Stage**	
**Cases**	**Age (Range) (IQR)**	**Ductal**	**Lobular**	**Other**	**I/II**	**III/IV**
Incident *n*=1558	52 (26–69) (47–58)	1075 (71%)	246 (16%)	186 (12%)	1431 (95%)	72 (5%)
Prevalent *n*=743	48 (25–54) (43–51)	540 (73%)	107 (14%)	94 (13%)	668 (95%)	38 (5%)
All *n*=2271	51 (25–69) (45–55)	1615 (72%)	353 (16%)	280 (12%)	2099 (95%)	110 (5%)

Characteristics of cases. Age: average age; range: (lowest–highest); IQR: interquartile range (the mid 50% of the distribution). For the 2280 controls the average age, age range and IQR were: 65 (44–81) (59–71).

**Table 3:** Top 10 two-way interactions from logistic regression

**Loci**	**df**	**LRT**	***P*-value**
TRX1-14 and TRXR2-04	8	14.77	0.00026
GPX4-02 and TRX1-14	8	14.20	0.00040
TRX1-06 and TRX1-14	8	11.96	0.0024
SOD1-01 and TRX1-06	8	11.71	0.0029
GPX4-06 and TRX1-14	8	10.34	0.0081
CAT-03 and SOD1-01	7	9.456	0.0085
CAT-01 and CAT-04	7	9.448	0.0085
GPX4-02 and TRXR2-04	8	10.10	0.0097
SOD1-01 and TRX1-14	8	9.868	0.011
GPX4-02 and TRX1-06	8	9.854	0.012

The ten two-way combinations with lowest *P*-value from the logistic regression (in descending order with the most significant first). The log-likelihood ratio statistic (LRT) is shown with degrees of freedom (df) and naïve *P*-value.

**Table 4:** Top 10 three-way interactions from logistic regression

**Loci**	**df**	**LRT**	***P*-value**
GPX4-02, TRX1-14 and TRXR2-04	26	29.64	0.00021
TRX1-14, TRX1-15 and TRXR2-04	25	28.68	0.00024
CAT-08, GSR-18 and SOD1-02	26	29.36	0.00025
TRX1-14, TRXR2-01 and TRXR2-04	26	29.10	0.00029
GSR-12, TRX1-14 and TRXR2-04	22	25.96	0.00032
SOD1-02,TRXR2-04 and TRXR2-08	26	28.52	0.00042
GPX1-01, GPX4-06 and TRXR2-03	26	28.51	0.00042
TRX1-14, TRX1-16 and TRXR2-10	23	26.29	0.00042
TRX1-14, TRXR2-04 and TRXR2-09	19	23.08	0.00047
GSR-16, SOD2-02 and TRXR2-05	26	28.04	0.00055

The 10 three-way combinations with lowest *P*-value from the logistic regression (in descending order with the most significant first). The log-likelihood ratio statistic (LRT) is shown with degrees of freedom (df) and naïve *P*-value.

**Table 5:** Genotype frequencies and effect sizes for most significant two-way interaction with logistic regression

	**TRXR2-04**			
	**0**	**1**	**2**	**Total**
TRX1-14				
0	362/441	700/622	291/292	1353/1355
	1 (N/A)	0.73 (0.61–0.87)	0.82 (0.67–1.02)	1 (N/A)
1	226/196	388/365	178/121	792/682
	0.71 (0.56–0.90)	0.77 (0.63–0.94)	0.56 (0.43–0.73)	0.86 (0.76–0.98)
2	37/37	57/66	19/34	113/137
	0.82 (0.51–1.32)	0.95 (0.65–1.39)	1.47 (0.82–2.62)	1.21 (0.92–1.58)
Total				
	625/674	1145/1053	488/447	
	1 (N/A)	0.85 (0.74–0.98)	0.85 (0.72–1.01)	

Genotype frequencies and effect sizes for most significant two-way interaction with logistic regression. Loci: TRX1-14 and TRXR2-04. At each locus: 0=common homozygote, 1=heterozygote and 2=rare homozygote. In each cell: controls/cases; odds ratio (95% confidence interval). The log-likelihood ratio statistic is 14.77 (*P*=0.00026, 8 df).

**Table 6:** Top 10 two-way interactions from multifactor dimensionality reduction

**Loci**	**Classification error**
GSR-16 and TRXR2-05	0.4566
TRX1-14 and TRXR2-04	0.4571
GPX1-01 and TRX1-14	0.4571
TRX1-14 and TRXR2-05	0.4577
TRXR2-01 and TRXR2-03	0.4587
TRX1-14 and TRXR1-03	0.4588
GPX4-04 and TRXR2-01	0.4590
GPX4-04 and TRX1-14	0.4590
TRX1-12 and TRXR2-05	0.4593
TRX1-14 and TRXR2-10	0.4594

The 10 two-way combinations with the lowest classification error for the whole data set are illustrated. The combinations are shown in descending order, that is, the combination with lowest classification error is first.

**Table 7:** Top 10 three-way interactions from multifactor dimensionality reduction

**Loci**	**Classification error**
GSR-16, TRX1-14 and TRXR2-05	0.4459
GPX4-02, TRX1-14 and TRXR2-04	0.4465
GSR-16, TRX1-06 and TRXR2-05	0.4484
SOD2-05, TRX1-11 and TRXR2-03	0.4486
TRX1-11, TRXR2-03 and TRXR2-12	0.4486
GPX4-04, TRXR2-01 and TRXR2-03	0.4488
GPX1-01, GSR-16 and TRXR2-05	0.4489
TRX1-11, TRXR2-01 and TRXR2-10	0.4489
TRXR2-01, TRXR2-03 and TRXR2-10	0.4490
CAT-03, TRX1-09 and TRXR2-03	0.4491

The 10 three-way combinations with the lowest classification error for the whole data set are illustrated. The combinations are shown in descending order, that is, the combination with lowest classification error is first.

**Table 8:** Genotype frequencies and effect sizes for most significant two-way interaction with multifactor dimensionality reduction

	**TRXR2-05**			
	**0**	**1**	**2**	**Total**
GSR-16				
0	748/701	596/505	108/125	1452/1331
	1 (N/A)	0.90 (0.77–1.06)	1.24 (0.94–1.63)	1 (N/A)
1	378/363	274/302	67/66	719/731
	1.02 (0.86–1.22)	1.18 (0.97–1.43)	1.05 (0.74–1.50)	1.11 (0.98–1.26)
2	55/32	24/39	8/5	87/76
	0.62 (0.40–0.97)	1.73 (1.03–2.91)	0.67 (0.22–2.05)	0.95 (0.69–1.32)
Total				
	1181/1096	894/846	183/196	
	1 (N/A)	1.02 (0.90–1.16)	1.15 (0.93–1.43)	

Genotype frequencies and effect sizes for most significant two-way interaction with multifactor dimensionality reduction. Loci: GSR-16 and TRXT2-05. At each locus: 0=common homozygote, 1=heterozygote and 2=rare homozygote. In each cell: Controls/cases; odds ratio (95% CI).

**Table 9:** Genotype frequencies and effect sizes for a two-way interaction from a permuted data set that was significant at the 5% threshold with logistic regression

	**B**			
	**0**	**1**	**2**	**Total**
A				
0	373/355	660/762	359/344	1392/1461
	1 (N/A)	1.21 (1.01–1.45)	1.01 (0.82–1.24)	1 (N/A)
1	183/102	294/291	148/135	625/528
	0.59 (0.44–0.78)	1.04 (0.84–1.29)	0.96 (0.73–1.26)	0.80 (0.70–0.92)
2	14/12	27/37	15/17	56/66
	0.90 (0.41–1.97)	1.44 (0.86–2.41)	1.19 (0.59–2.42)	1.12 (0.78–1.62)
Total				
	570/469	981/1090	522/496	
	1 (N/A)	1.35 (1.16–1.57)	1.15 (0.97–1.37)	

Genotype frequencies and effect sizes for a two-way interaction from a permuted data set that was significant at the 5% threshold with logistic regression. Loci: A and B. At each locus: 0=common homozygote, 1=heterozygote and 2=rare homozygote. In each cell: Controls/cases; odds ratio (95 % confidence interval). The naïve *P*-value is 0.000044 (8 df) and the log-likelihood ratio statistic is 33.81. The data set was picked out based on the naïve *P*-value, which is the threshold naïve *P*-value for the 5% tail of the empirical null distribution.
